# Enhanced selective capture of phosphomonoester lipids enabling highly sensitive detection of sphingosine 1-phosphate

**DOI:** 10.1007/s00216-023-04937-8

**Published:** 2023-09-22

**Authors:** Giuliana Grasso, Eduardo M. Sommella, Fabrizio Merciai, Rahma Abouhany, Sudhirkumar A. Shinde, Pietro Campiglia, Börje Sellergren, Carlo Crescenzi

**Affiliations:** 1 Department of Pharmacy, University of Salerno, Via Giovanni Paolo II 132, 84084 Fisciano, SA Italy; 2 Biofilm Research Center for Biointerfaces, Department of Biomedical Sciences, Faculty of Health and Society, Malmö University, 23014 Malmö, Sweden; 3 School of Consciousness, Dr. Vishwanath Karad MIT World Peace University, 411038 Pune, India

**Keywords:** Bioanalytical methods, Biological samples, Biopolymers/lipids, Clinical/biomedical analysis, Pharmaceuticals, Polymers

## Abstract

Sphingolipids play crucial roles in cellular membranes, myelin stability, and signalling responses to physiological cues and stress. Among them, sphingosine 1-phosphate (S1P) has been recognized as a relevant biomarker for neurodegenerative diseases, and its analogue FTY-720 has been approved by the FDA for the treatment of relapsing–remitting multiple sclerosis. Focusing on these targets, we here report three novel polymeric capture phases for the selective extraction of the natural biomarker and its analogue drug. To enhance analytical performance, we employed different synthetic approaches using a cationic monomer and a hydrophobic copolymer of styrene-DVB. Results have demonstrated high affinity of the sorbents towards S1P and fingolimod phosphate (FTY-720-P, FP). This evidence proved that lipids containing phosphate diester moiety in their structures did not constitute obstacles for the interaction of phosphate monoester lipids when loaded into an SPE cartridge. Our suggested approach offers a valuable tool for developing efficient analytical procedures.

**Graphical Abstract:**

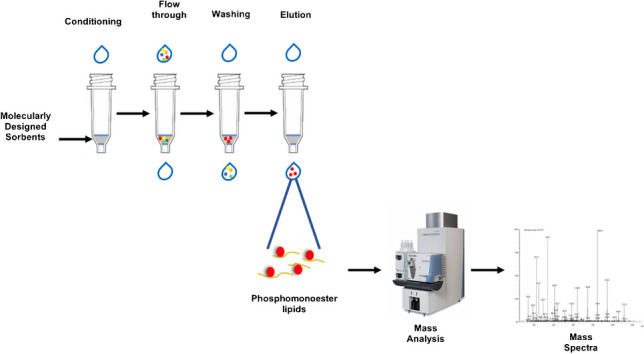

**Supplementary Information:**

The online version contains supplementary material available at 10.1007/s00216-023-04937-8.

## Introduction

**Fig. 1:**
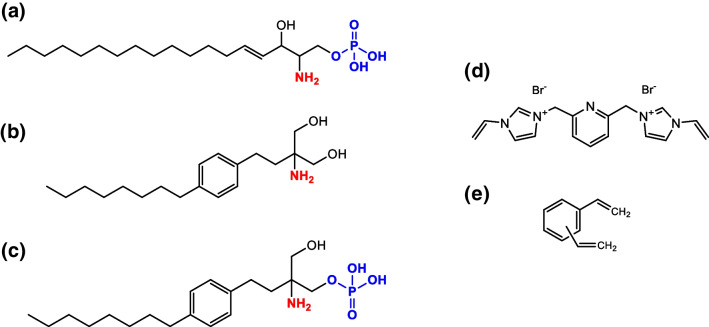
Chemical structures of **a** sphingosine 1-phosphate, **b** fingolimod, **c** fingolimod phosphate, **d** bis-imidazolium functional monomer, and **e** divinylbenzene cross-linker

Sphingolipids, in particular glycosphingolipids, are common constituents of plasma membranes, especially embedded in neural cells [1]. In fact, they represent an important class of lipids, considered as units in membranes’ building blocks and components of myelin, a fundamental substance that covers nervous fibres and allows neurons connection. The simple physiological metabolite of the sphingosine pathway, *sphingosine 1-phosphate* (S1P) (Fig. 1a), performs a plethora of functions and has been recognized as a relevant biomarker involved in cardiovascular diseases [2], in inflammation process, in cancer development, and in neurodegenerative diseases. S1P is formed intracellularly by sphingosine phosphorylation (derived from ceramide deacylation), a process catalysed by the two sphingosine kinases SPHK1 and SPHK2. The extruded S1P can behave as a signalling molecule not only via the coupling to five specific G-protein-coupled receptors (S1P receptor 1 (S1PR1) to S1PR5), but also by the activation of intracellular pathways, before going towards its metabolism operated by S1P lyase [3]. Kunkel G. T. et al. defined for the first time the “S1P” axis referring to the functions of the signalling molecule S1P, its receptors, and intracellular targets, as well as the proteins that synthesize, transport, and degrade S1P. Each of these steps could be therapeutically targeted [4, 5]. With this purpose, specific S1PR agonists and antagonists, of first- and second-generation, have been developed. The most used pharmaceutical preparation is fingolimod (Fig. 1b), an immunomodulating drug commercially referred as Gilenya™ (Novartis) [6, 7]. The drug has been clinically approved for the treatment of relapsing and remitting multiple sclerosis in the USA and Europe [8]. Fingolimod is a sphingosine analogue (not containing the phosphate group) that is phosphorylated primarily by SPHK2 to form *phosphorylated fingolimod* (FP) (Fig. 1c), which is an agonist at all the S1PRs except for S1PR2 [9, 10]. The increased relevance and physio-pathological significance of S1P in clinical practice were up-streamed in the last 20 years and determined an up-tick in the optimization of the analytical processes that bring to the identification and quantification of this potential biomarker [11–18].

The search for novel biomarkers requires adequate tools for accurately quantifying a broad range of potential target analytes. The complexity of biological matrices, being obstacles for the development of effective diagnostic tools, might be overstepped by means of efficient class-selective sorbents. The phenomenon of matrix effect is of primary concern in bioanalysis when ESI LC–MS detection is used for quantitative purpose. The presence of co-eluted compounds, affecting pH, ionic strength, and surface tension or competing for the ionization process, may strongly affect the ionization yield of target analytic compounds, thus requiring the use of expensive, and not always available, isotopically labelled internal standards. Because of the relevance of this issue, the US FDA explicitly recommends to investigate the occurrence of such effects when developing analytical methods [19]. Selective extraction of target analytes might reduce the matrix effects resulting in more accurate quantitative determination. Methods based on the molecular recognition, such as solid-phase extraction (SPE) [20], immuno-affinity extraction (IAE) [21], and molecularly imprinted polymers (MIPs) [22], have been developed and could be considered promising approach to identify and quantify specific biomarkers. Inspired by host–guest chemistry, monomers binding specific substructures of the target have been used to generate artificial receptors capable of class-selective trapping of compounds. Since the majority of biofluids consists of high percentage of water, a protic polar solvent that can form hydrogen bonds, the host monomer selected for this study was the bis-imidazolium (Fig. 1d), a charged receptor that can take advantage of the electrostatic effect and thus compete more effectively with polar protic solvents. In fact, imidazolium-based receptors can interact with anions through (C-H)^+^–-X^−^ type of ionic hydrogen bond [23] and are especially potent receptors for oxyanions [24, 25]. More recently, it has been reported that copolymers based on bis-imidazolium and cross-linked by means of ethylene glycol dimethacrylate (EGDMA) per se exhibited strong affinity for phosphomonoesters [26, 27], lending them useful as capture phases for deep profiling of S1P isomers [28].

**Fig. 2:**
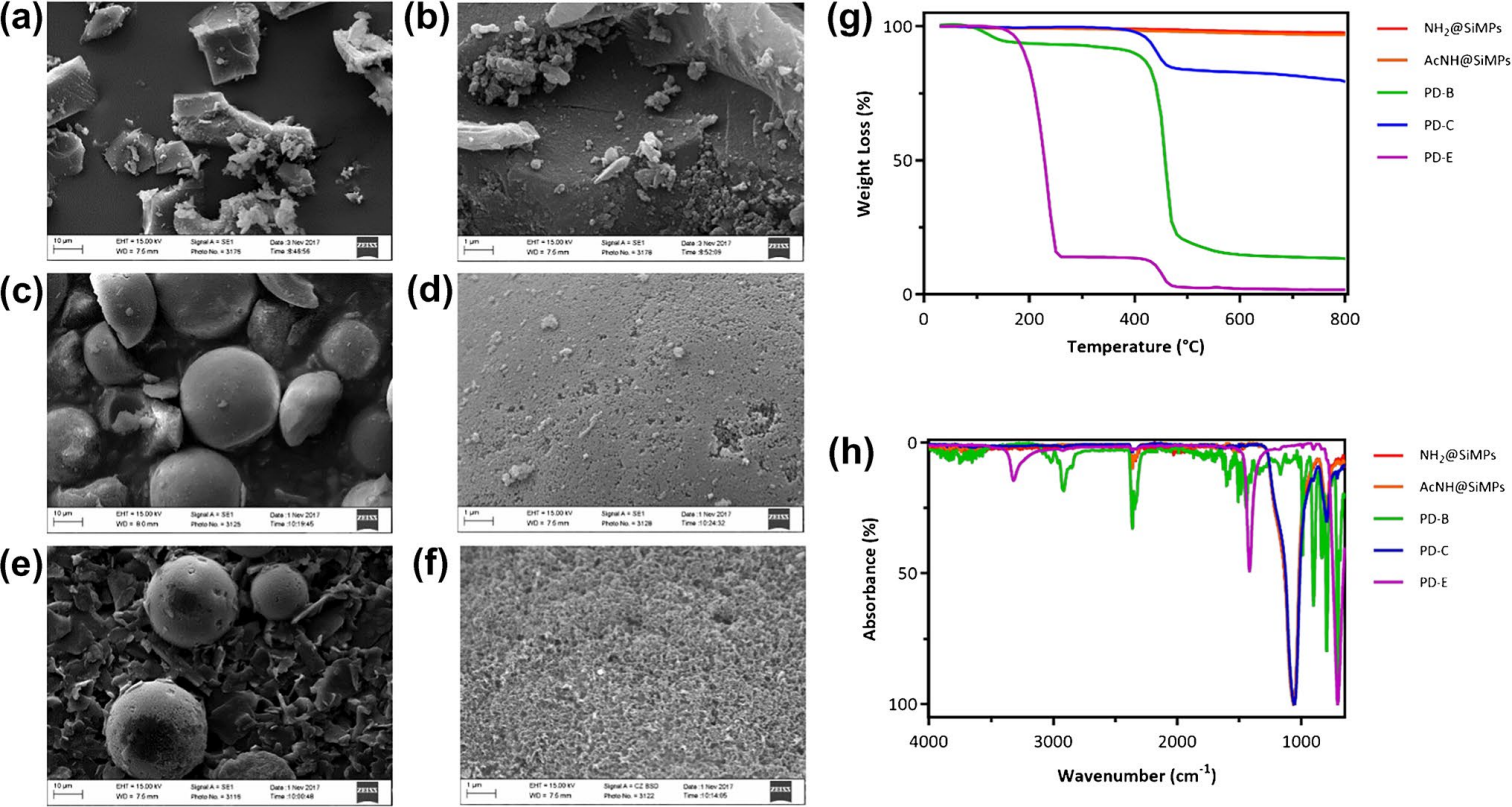
Morphological and physico-chemical characterizations: SEM micrographs of **a**–**b** PD-B, **c**–**d** PD–C, and **e**–**f** PD-E at 10 × and 25 × magnifications; **g** thermographs and **h** FT-IR spectra of PD-B, amino-functionalized silica microspheres, acetylated-modified silica template, PD–C and PD-E template, PD–C, and PD-E

Building on our previous study, the aim of this work was to optimize bis-imidazolium-based capture materials cross-linked by divinilbenzene (DVB) (Fig. 2e) for direct enrichment of S1P and FP from highly competitive biological matrices.

## Materials and methods

### Chemicals

2,6-Bis-(bromomethyl)pyridine, *N*-vinylimidazole, sulphur, acetonitrile anhydrous (99,8%), acetic acid (Ac. A), trifluoroacetic acid (TFA), phosphoric acid (PA), phenyl phosphonic acid (PPA), 2,5-dihydroxybenzoic acid (DHB), and ammonium formate for mass spectrometry (≥ 99.0%) were purchased from Sigma-Aldrich (Steinheim, Germany). Divinylbenzene (DVB) was purchased from Sigma-Aldrich (Steinheim, Germany) and was purified by filtration through a short column of neutral aluminium oxide prior to use. Ninhydrin were from Sigma-Aldrich (Milwaukee, USA). Dry dimethylformamide (dry DMF) was from Acros Organics. Acetic anhydride (Ac_2_O) and formic acid (FA) were from Fluka (Deisenhofer, Germany). N,N’-Azo-bis-(2,4-dimethyl)valeronitrile (ABDV) was purchased from Wako Chemicals GmbH (Neuss, Germany). Methanol anhydrous 99.9% (dry MeOH), acetonitrile (ACN) ≥ 99.8% HiPerSolv CHROMANORM® Reag. Ph. Eur., gradient grade for HPLC, methanol (MeOH) ≥ 99.8% HiPerSolv CHROMANORM® Reag. Ph. Eur., gradient grade for HiPerSolv CHROMANORM® Reag. Ph. Eur., gradient grade for HPLC, and dimethyl sulphoxide-d_6_ (DMSO-d_6_) were from VWR chemicals (Radnor, PA, USA). All additives and mobile phases were LC–MS grade and purchased from Sigma-Aldrich (Milan, Italy). Amino-functionalized silica microparticles (NH_2_@SiMPs), with an average particle size of 20–45 μm, a surface area (*S*) of 45m^2^ g^−1^, average pore diameter (*D*_*p*_) of 47.5 nm, and a pore volume (*V*_*p*_) of 0.81 mL g^−1^, were purchased from Fuji Silysia Chemical Ltd. (Kozoji-cho, Kasugai Aichi, Japan). 

### Lipid standards and biological samples

Sphingosine 1-phosphate (d18:1) (S1P), 1,2-dimyristoyl-*sn*-glycero-3-phospho-(1’-rac-glycerol) sodium salt (DMPG), 1,2-dimyristoyl-*sn*-glicero-3-phosphatidylcoline (DMPC), 1,1’,2,2’-tetramyristoyl cardiolipin sodium salt (CL), 1,2-dimyristoyl-*sn*-glycero-3-phospho-L-serine sodium salt (DMPS), and 1,2-dimyristoyl-sn-glycero-3-phosphoethanolamine (DMPE) were purchased from Avanti Polar Lipid Inc. (Alabaster, Alabama, USA). 1,2-Dipalmitoyl-*sn*-glycero-3-phosphatidylcholine-D62 was purchased by Larodan (Solna, Sweden). Fingolimod (FTY720) and fingolimod phosphate (FTY720-P, FP) were purchased from Novartis Institutes for BioMedical Research (Basel, Switzerland). More details about the lipids used in competitive binding experiments are listed in the Electronic Supplementary Material Table S1. Commercial human serum from human male AB plasma, USA origin, sterile-filtered, was purchased from Sigma-Aldrich (Steinheim, Germany). Human blood for plasma was obtained from healthy volunteer donors recruited from healthcare workers at the University Hospital “San Giovanni di Dio e Ruggi d’Aragona” and treated using EDTA as an anticoagulant. Blood samples were centrifuged at 2200 × g for 15 min to isolate plasma and stored at − 80 °C within 45 min of blood collection.

### Synthetic procedures

#### Synthesis of bis-imidazolium-based functional monomer

The functional monomer *1-vinyl-3-{3-[(1-vinyl-1H-imidazol-3-ium-3-yl)methyl]benzyl}-1H-imidazol-3-ium dibromide* (Fig. 1d) was prepared as reported previously [27]. Briefly, a solution of 2,6-bis-(bromomethyl) pyridine (4 g, 15.09 mmol) and *N*-vinylimidazole (2.755 mL, 30.42 mmol) in dry ACN (200 mL) was refluxed overnight, at 100 °C, with a spatula tip of sulphur to avoid polymerization. After cooling down at room temperature, the reaction mixture was dried under vacuum, and the final product was obtained through solubilization in ethanol and precipitation in diethyl ether, with yield of 83%. The purity of monomer was characterized by ^1^H and ^13^C NMR spectroscopy, and the data was matched with our published report [27].

#### Amino-functionalized silica modification (AcNH@Si)

NH_2_@Si (20 g) was suspended in DMF (100 mL) and stirred in a 250-mL round bottomed flask. Then, acetic anhydride was added (20 mL), and the suspension was stirred at room temperature overnight. Silica particles obtained after reaction were filtered off, washed with DMF (3 × 50 mL) and MeOH (3 × 50 mL), and dried in oven at 35 °C overnight to yield N-acetylated silica (AcNH@Si). Ninhydrin test, performed after modification, resulted negative and confirmed complete functionalization of silica amino groups.

#### Bulk copolymer s (PD-B and PE-B)

Bis-imidazolium-based functional monomer (0.780 mmol, 358 mg), DVB (30 mmol, 4.317 mL), and ABDV (1.5% w/w of total monomers) were dissolved in 1.060 mL solution of anhydrous MeOH/Toluene (1:1, v/v) in a 20-mL glass vial, and the mixture was purged with N_2_ at room temperature for 15 min. Subsequently, the mixture was heated at 45 °C to allow starting the polymerization reaction for 24 h, and then the temperature was increased till 80 °C for other 2–3 h. The synthesized bulk polymer was crushed and then transferred into a 50-mL centrifuge tube and washed three times with a solution of MeOH/1 M aq. HCl (1:1, v/v) and three times with pure MeOH. The particles were then dried in oven at 60 °C overnight. The particles were crushed again and sieved, obtaining three different particle sizes: ≥ 50 μm, 50–25 μm, and ≤ 25 μm. For our analysis and purpose, we used particles in the size range 25–50 μm.

#### Composite sorbents (PD–C and PE-C)

A pre-polymerization mixture was first prepared as follows. In a 20-mL glass vial, bis-imidazolium functional monomer (0.210 mmol, 100 mg) and DVB (8.4 mmol, 1.173 mL) were dissolved in 0.511 mL of the porogen mixture MeOH/Toluene (1:1, v/v). Then the initiator ABDV (1.5 w/w % of the total monomers) was added to the solution, and the pre-polymerization mixture was purged under a flow of N_2_ for 15 min. In another 20-mL glass vial, AcNH@Si500 (2.2 g) were de-aerated and purged with a continuous flow of N_2_ and then allowed to soak the pre-polymerization mixture until particles were freely flowing. The tube was then sealed, and the polymerization was started by placing the tube in an oven heated at 50 °C for 24 h. The vial was then kept at 70 °C for 4 h. The resulting polymer was transferred in a 50-mL polypropylene centrifugations tubes and washed with a solution of MeOH/1 M aq. HCl (8/2 v/v) (3 × 50 mL). The solvent was then extracted in a Soxhlet apparatus with MeOH for 24 h. The resulting composite particles were then dried under vacuum overnight.

#### Etched particles (PD-E and PE-E)

A part of the composite microspheres (1 g ca) were transferred into a 50-mL centrifugation tube, and 40 mL of etching solution (NH_4_HF_2_ 3 M prepared in H_2_O) was added. The polymer was shaken for 24 h on a rocking plate. The resulting material was transferred in a 50-mL polypropylene centrifugation tubes and washed with a solution of MeOH/1 M aq. HCl (8/2 v/v), for at least three times (3 × 50 mL). The solvent was then extracted in a Soxhlet apparatus with MeOH for 24 h. The resulting etching particles were then dried under vacuum overnight.

### Methods

#### Batch binding test

To estimate the sorbent affinity, 10 mg of each synthesized material was separately incubated in 1 mL of methanol containing PPA at 1, 0.75, 0.5, 0.25, 0.1, 0.075, 0.05, and 0.025 mM and shaken on a rocking plate for 12 h, at room temperature. Consequently, all the samples were centrifuged, and 500 μL of supernatants was analysed by C18-HPLC–UV analysis.

#### Sample preparation

Stock solutions of standards were prepared in MeOH to obtain a concentration of 1 mg/mL for sphingosine 1-phosphate (d18:1) (S1P), fingolimod (FTY-720), fingolimod phosphate (FTY- 720 P), 1,2-dimyristoyl-*sn*-glycero-3-phospho-(1’-rac-glycerol) sodium salt (DMPG), 1,2-dimyristoyl-*sn*-glicero-3-phosphatidylcoline (DMPC), 1,1’,2,2’-tetramyristoyl cardiolipin sodium salt (CL), 1,2-dimyristoyl-sn-glycero-3-phospho-L-serine (sodium salt) (DMPS), and 1,2-dimyristoyl-sn-glycero-3-phosphoethanolamine (DMPE). Then, the stock solutions were further diluted in a concentration range of 0–150 μM using methanol.

#### Human plasma sample preparation

For lipid extractions, 100 μL of human plasma was added to 1 mL of MeOH enriched with 10 nmol/mL S1P, FTY720, and FTY720-P. The mixture was vortexed for 30 s, sonicated for 30 min, and centrifuged at 14,000 rpm, at 4 °C, for 10 min to allow plasma protein precipitation. The supernatant was transferred into fresh tubes. Loading fractions were then prepared in 1-mL volume diluting 100 μL of plasma lipid extract in 2-propanol (for extractions on bulk resin) and in ACN (for extractions using composite sorbent and etched materials).

#### Human serum sample preparation 

Commercial human serum (Human Male AB plasma, USA origin, sterile-filtered, Sigma-Aldrich) was firstly thawed. A stock solution was prepared by diluting human serum in water in the ratio 1:20. For lipid extractions, 20 mL of MeOH was added to the stock solution of human serum. The mixture was vortexed for 30 s, sonicated for 30 min, and centrifuged at 14,000 rpm at 4 °C for 10 min to allow further protein precipitation. The final supernatant was transferred into a new fresh tube. Loading fractions were then prepared in 1-mL volume diluting 100 μL of human serum lipid extract in 2-propanol (for bulk extractions) and in ACN (for composite material).

### Lipid mixture for competitive SPE recovery experiments

The lipid mixture used for competitive SPE recovery experiments was as follows: sphingosine 1-phosphate (S1P); fingolimod phosphate FTY720-P (FP); fingolimod FTY720 (F); 1,2-dimyristoyl-sn-glycero-3-phosphorylglycerol sodium salt (DMPG); 1,2-dimyristoyl-sn-glycero-3-phosphocholine (DMPC); 1,2-dimyristoyl-sn-glycero-3-phosphoethanolamine (DMPE); 1,2-dimyristoyl-sn-glycero-3-phosphoserine sodium salt (DMPS); and 1,1’,2,2’-tetramyristoyl cardiolipin sodium salt (CL). More details are in Electronic Supplementary Material.

#### Solid-phase extraction (SPE) protocols

One hundred milligramme of each polymer was packed into SPE cartridges using on the top and at the bottom filters with 10-μm pore size (Mobicol Classic, MoBiTec). For bulk material (particles size range 25–50 μm), the cartridge was equilibrated with 3 × 1 mL 2-propanol. The loading fraction was passed through the cartridge twice. The washing steps were executed with 1 mL 2-propanol and 1 mL 2-propanol/MeOH (1/1, v/v). The elution fractions were collected with 2 × 0.8 mL MeOH/CHCl_3_ (1/1, v/v) plus 1% TFA. All fractions were then dried under vacuum and reconstituted in 100 μL MeOH, sonicated for 10 min, and transferred into insert vial for LC–MS analysis. For the composite sorbent and the etched material, the cartridge was conditioned using 3 × 1 mL regeneration buffer MeOH/acetic acid/H2O (6/3/1, v/v/v), 3 × 1 mL 2-propanol, and 3 × 1 mL ACN. Next, the loading fraction was passed through the cartridge twice. The washing steps were performed with 1 mL ACN, 2 × 1 mL 2-propanol. The elution fractions were collected three times by adding 0.8 mL MeOH plus 0.1% TFA. All fractions were then dried under vacuum and reconstituted in 100 μL MeOH sonicated for 10 min and transferred into insert vial for LC–MS analysis. Cartridges prepared have been reused after regeneration step (3 × 1 mL MeOH/acetic acid/H2O (6/3/1, v/v/v)) and equilibration to obtain experiments in triplicate.

### Instrumentation

#### Optical microscopy 

Optical micrographs were acquired using Nikon Optiphot epifluorescence microscope equipped with polarizing filters, phase contrast, and a DS-U1 digital camera.

#### Scanning electron microscopy (SEM) 

The particle morphology, size, and size distribution were determined using Zeiss EVO LS 10 CANSEM (Carl Zeiss AG, Oberkochen, Germany) at T ¼ 25 °C, EHT ¼ 15 kV, and WD ¼ 4.5 mm. For imaging, particles were covered by a thin layer of gold.

#### Nitrogen sorption by means of Brunauer–Emmett–Teller (BET)

Nitrogen sorption measurements were performed on the Nova 4200*e* Sorption analyser (Quantachrome Instruments, USA). The specific surface areas *S* were evaluated by using the Brunauer–Emmett–Teller (BET) method.

#### Thermogravimetric analysis (TGA)

TGA was carried out through TGAQ500 (TA Instruments). Ca 10 mg of each sample was placed in a platinum pan, after tare. The sample was heated at 10 °C/min till 800 °C, under N_2_ atmosphere.

#### Fourier-transform infrared spectroscopy (FT-IR)

FT-IR spectra were recorded using a Thermo Nicolet Nexus 6700 instrument (Thermo Scientific, Waltham, MA, USA).

#### High-performance liquid chromatography coupled to UV/Vis spectroscopy (HPCL-UV/Vis)

Quantitative analysis of PPA for batch binding tests was performed using HPLC analysis by Alliance 2795 instrument (Waters, Milford, MA, USA) equipped with 2996 PDA detector (Waters, Milford, MA, USA). The stationary phase was a RP Luna C18 column, 100 Å, 250 × 4.6 mm, 5-μm particle size (Phenomenex, California, US), and the mobile phase prepared was H_2_O/MeOH (68:32). The method lasted 15 min at a flow rate of 0.600 mL/min. The injection volume was 20 μL, and the UV detector was set up at 225 nm.

#### Matrix-assisted laser desorption ionization–time-of-flight mass spectrometry (MALDI-TOF–MS) 

For fast evaluation of binding abilities, MALDI-TOF analysis was performed (see Electronic Supplementary Material Fig. S1). All mass spectra were obtained using an Ultra-fleXtreme MALDI-TOF/TOF MS/MS (Bruker Daltonics, Bremen, Germany) controlled by flexControl software (version 2.4, Bruker Daltonics, Bremen, Germany). The system was set up in positive ion linear mode in the m/z range of 200–1400. The matrix used in the experiment was a solution of 2,5-dihydroxybenzoic acid (DHB, 20 mg/mL) in 50% acetonitrile (ACN), 0.1% trifluoroacetic acid (TFA), and 1% phosphoric acid (PA). Relative laser power was set at 60%. Signals came from an average of 1000 laser shots (5 × 200 shots). Samples were spotted on MTP 384 target plate polished steel BC (# 8,280,781).

#### Ultra-high-performance liquid chromatography coupled to tandem mass spectrometry (UHPLC-MS/MS) analysis

Chromatographic analysis was carried out with a Shimadzu Nexera (Shimadzu, Milan, Italy) UHPLC consisting of two LC 30 AD pumps, a SIL 30AC autosampler, a CTO 20AC column oven, and a CBM 20 A controller, and the system was coupled online to a triple quadrupole LC–MS 8050 (Shimadzu, Kyoto, Japan) equipped with an ESI source. The separation was performed on a Kinetex EVO C18, 100 Å, 150 × 2.1 mm × 2.6 µm, at a flow rate of 0.5 mL/min, employing as mobile phase A, ACN/water, 60/40 10 mM containing HCOONH_4_ and 0.1% HCOOH (v/v%), and B isopropanol/ACN 90/10 plus 0.1% HCOOH (v/v%) with the following gradient, 0.0–2.0 min, isocratic at 0% B, 2.01–4.50 min, 0–50% B, 4.51–8.00 min 50–80% B, 8.01–9.00 min, 80–99% B, and isocratic for 1.50 min. Returning to 0% in 4.50 min, 2 mL was injected. All additives and mobile phases were LC–MS grade and purchased from Sigma-Aldrich (Milan, Italy). MS/MS analysis of lipids was conducted in multiple reaction monitoring (MRM). The ESI was operated both in negative and positive ionization setting the following parameters, interface temperature 300 °C, desolvation line temperature 250 °C, and heat block temperature 400 °C, and nebulizing gas, drying gas and heating gas were set to 3, 10, and 10 L/min. Detailed description of the MS/MS detection is reported in “Supplementary Information”. All the UHPLC-MS/MS analyses were from triplicate experiment, and the result of each experiment was determined by duplicated chromatographic runs separated by injection of blank solvents.

For data analysis, mass spectrometric data analysis was performed using FlexAnalysis 3.4 software (Bruker Daltonik GmbH, Bremen, Germany). Plots and calculations were performed using GraphPad Prism (GraphPad Software, San Diego, California USA).

## Results and discussion

### Polymer preparation and characterizations

Considerable effort has been dedicated to developing capture phases that exhibit selective binding to S1P [27–29]. In this study, our primary objective was to compare the moderately polar EGDMA cross-linked materials with the more hydrophobic DVB-based sorbent with respect to their ability to retain S1P and the immunomodulatory active drug phosphorylated FP from biological fluid samples. Additionally, we capitalized on our previously reported templating strategy, which yields spherical beads with controlled pore size and enhanced mass transfer properties. The synthesized promising DVB-based materials were morphologically investigated by scanning electron microscopy (SEM), and physico-chemical was characterized by means of TGA, FT-IR and nitrogen adsorption surface area analysis. The PD-B exhibited irregular shapes due to the crushing step and confirming the expected rough surface (Fig. 2a–b). A different morphology was found for the composite PD–C and the templated materials PD-E prepared using the silica microspheres (d ≈ 25–40 µm) as template. As expected, PD–C showed a relatively smooth surface (Fig. 2c–d), whereas PD-E revealed pores (Fig. 2e–f), deriving from the removal of the silica template. The weight changes of PD-B, PD–C, and PD-E sorbents were determined through TGA analysis (Table 1). The thermographs shown in Fig. 2g indicated a significantly high final residue (approximately 80%) for PD–C after heating the material up to 800 °C, attributable to the presence of silica microparticles. Conversely, PD-B exhibited a relatively low residue of 13.23%. The remarkably low residue (2%) was observed for PD-E. Additionally, thermal stability analyses revealed a consistent loss of the amide terminal group at 245 °C for all the sorbents, followed by nearly complete degradation of the DVB polymer at 465 °C, ensuring the synthesized materials’ thermal stability. These results confirmed the functionalization of the silica pores in PD–C and the efficient removal of the silica template from PD-E.

**Table 1:** Properties of the synthesized DVB materials, binding parameter of Langmuir model (*B*_max_ and *K*_eq_), BET specific surface area S, and TGA weight loss (%) at 800 °C

Polymers	*Template*	*Format*	*B*_max_ (µmol/g)	*K*_eq_	*S* (m^2^ g^−1^)	Weight loss (%)
PD-B	/	Bulk	19.6	3.37 × 10^−03^	2.50	85.88
PD–C	SiMPs	Composite	7.6	1.04 × 10^−03^	96.60	20.45
PD-E	SiMPs	Etched	5.5	1.96 × 10^−03^	17.60	98.33

When analysed using ATR-FT-IR spectroscopy, the PD-B material revealed the characteristic bands at 2990 cm^−1^ (C-H stretching), 1600 cm^−1^ (vinyl -C = C- groups stretching), and various peaks in the fingerprint zone (500–400 cm^−1^, -CH_2_- rocking). The FT-IR spectra of the PD–C polymer (Fig. 2h) showed a strong and intense overlapping band at 1100 cm^−1^ (Si–O-Si stretching), which is a distinctive feature of the silica-supported material and its precursors (SiO_2_@NH_2_ and SiO_2_@NHAc microspheres). However, the PD-E polymer did not exhibit this band, confirming the complete removal of silica from the microspheres. Nevertheless, the PD-E sorbent displayed a weak band at 3220 cm^−1^ (= C-H stretching) and a sharp peak with medium intensity at 1470 cm^−1^ (O-CH_2_ bending), indicating the exposure of the bis-imidazolium within the pores of the silica spheres.

Finally, specific surface was determined by nitrogen adsorption experiment by means of Brunauer–Emmett–Teller (BET) method. The data reported in Table 1 showed a much more developed specific surface area of PD–C, while surprisingly PD-E displayed a lower surface area. These results could be directly linked to a partial collapse of the etched material structure resulting in a lower porosity level.

### Binding affinity

**Fig. 3:**
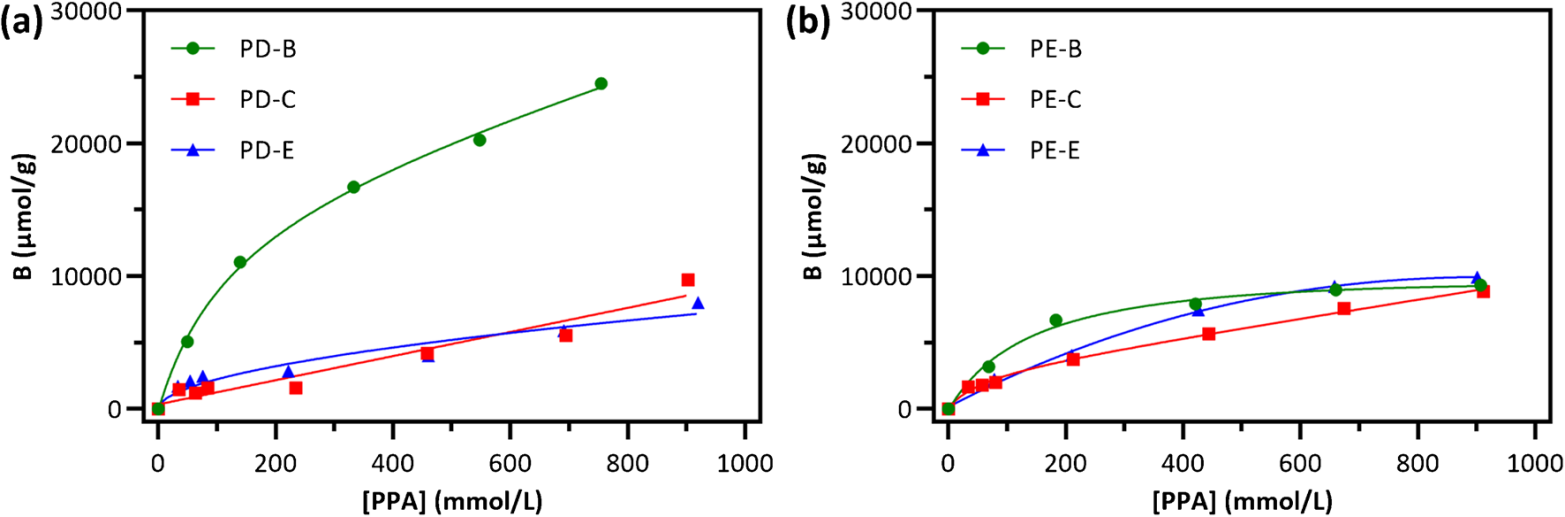
Binding isotherms of PPA (concentration range 0.025–1 mM) for DVB (**a**) and EGDMA-based polymers (**b**) in methanol calculated with nonlinear regression

To assess the binding affinity and the maximum capacity of the materials, batch adsorption experiments were conducted in methanol using phenylphosphonic acid (PPA) as a model ligand at concentration levels ranging from 0.1 to 1 mM. After equilibrium, the concentrations of PPA in the supernatant solution were determined by RP HPLC–UV analysis. Binding capacity B was calculated according to the following equation:

$$B=\frac{{B}_{max}{K}_{eq}{C}_{f}}{1+{K}_{eq}{C}_{f}}$$

where ${B}_{max}$ was the maximum binding capacity, ${K}_{eq}$ is the binding constant, and ${C}_{f}$ is the solute concentration detected in the supernatant solution. The binding isotherms (Fig. 3) were obtained using the nonlinear least square fitting, and the calculations were applied to experimental data according to Langmuir model. Binding parameters are reported in Table 1 and show an increased binding capacity for the materials prepared using DVB as cross-linker, in comparison to the EGDMA cross-linked materials, demonstrating that improved hydrophobicity of the polymers, coming from the employment of DVB as cross-linker, significantly increased non-selective adsorption of PPA. In such circumstances, for DVB-based materials, higher extraction efficiency might be combined with selective retention using proper conditions in the process neither led to an increase in the specific surface, neither improved sorbent affinity under the investigated conditions.

### Feasibility of SPE recovery experiments in real samples

**Fig. 4:**
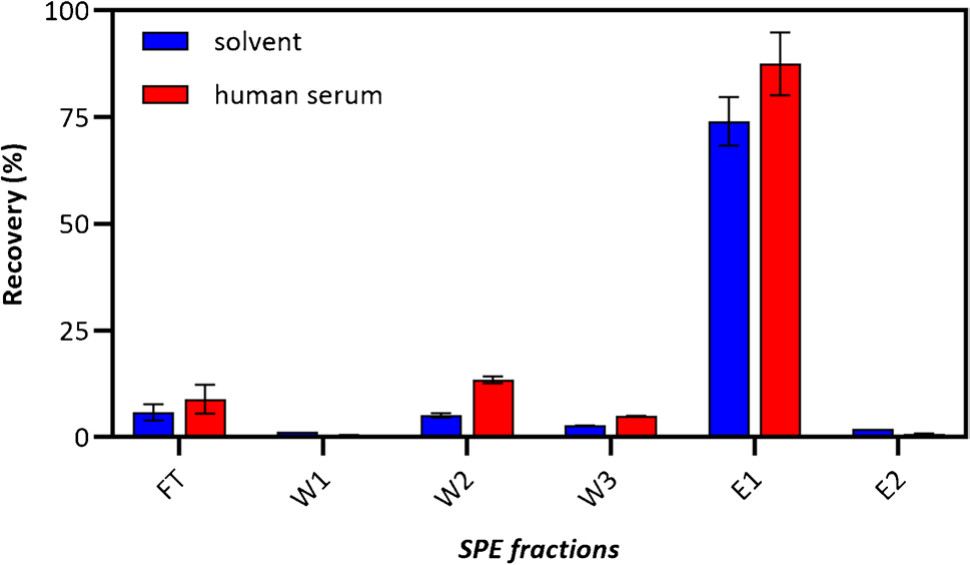
Extraction–elution curve of S1P on SPE cartridge with DVB-composite material. The data presented represents the mean average of three replicates, with error bars indicating the standard deviation

The potential use of the DVB-based polymers was tested by means of SPE cartridges to study the selective enrichment of phospholipids from real biological samples. A preliminarily evaluation was carried out by comparing the extraction and elution curves of S1P from 1 mL of spiked solvent IPA/MeOH (1/1, v/v) and 1 mL of spiked human serum sample. These experiments were performed using an SPE cartridge packed with 30 mg of PD–C materials and previously optimized extraction protocols [28–30]. The results, depicted in Fig. 4, demonstrated efficient retention of the phosphomonoester S1P lipid and rapid release of the analyte in the initial 0.8 mL fraction of the elution phase MeOH/CHCl3 (1/1, v/v) with 1% TFA. Moreover, results demonstrate that there were no significant differences in the sorbent’s performance when extracting and desorbing S1P from spiked solvents or spiked human serum samples.

**Fig. 5:**
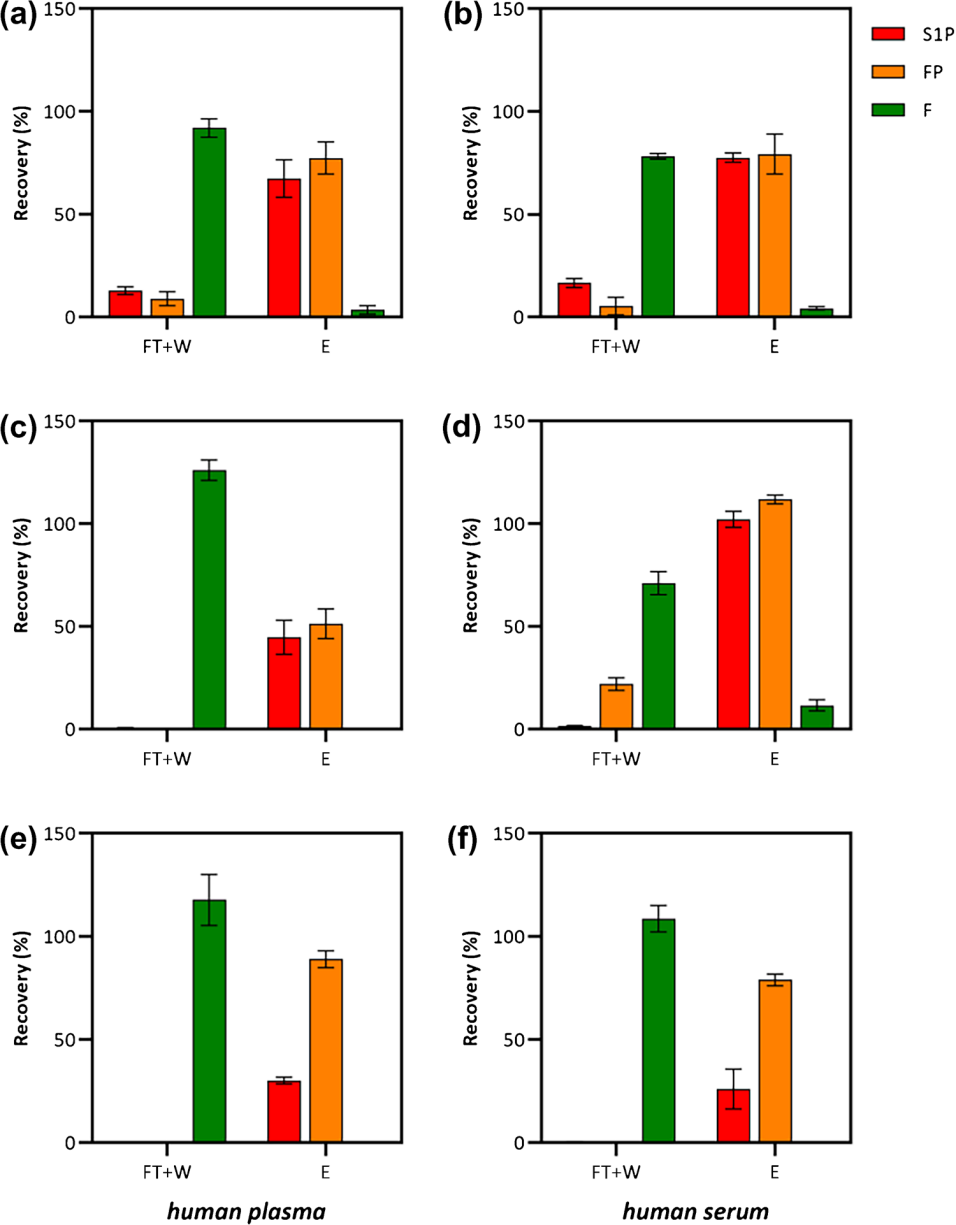
Recovery experiments from artificially spiked human plasma (right side) and serum (left side) samples performed using **a**, **b** PD-B, **c**, **d** PD–C, and **e**, **f** PD-E materials. Standard employed were S1P, FP, and F. FT + W: flow through + washing steps; E: elution steps. Data reported are the mean average of three replicas, and the error bars represents the standard deviation

The improved selectivity of the three DVB-based materials towards the selected target molecules was finally assessed by performing SPE experiments of S1P, FP, and F from spiked human plasma and spiked human serum samples. In order to verify the potential occurrence of competition/displacement effects on the binding sites, thus simulating a potential analytical application of the sorbents, these experiments were performed biological samples further fortified with a high concentration (10 μmol/L each) of selected phospholipids (composition of lipid mixture is reported in the experimental section). The results presented in Fig. 5 summarize the recovery percentages obtained from three replicate experiments. Remarkably, all sorbents demonstrated efficient and selective retention of the phosphomonoesters S1P and FP, with no relevant presence in the flow through + washing fractions (FT + W), while found in the elution steps (E). Conversely, the retention of the F prodrug was limited across the experiments, with the majority being released during the FT + W steps.

Figure 5a and b provided a detailed overview of the recovery experiments conducted on bulk PD-B from human plasma and human serum, respectively. For both the matrices, the LC–MS/MS analysis revealed approximately 90% for F in the FT + W fraction, and almost 80% for the phosphorylated forms of fingolimod and sphingosine in E. In contrast, the composite PD–C sorbent made registered dissimilar recovery results when employed for the extraction of the analytes from plasma samples (Fig. 5c) or from human serum samples (Fig. 5d). In particular, for human plasma and human serum SPE experiments, the bis-imidazolium monomer did not retain the F prodrug, which was found in both cases in the FT + W fractions. On the other hand, for human plasma samples, FP and S1P recovery was approximately 50% each. These outcomes should be ascribed not only to the complexity of the matrices used, but also to the architecture of the composite material developed. The composite adsorbent presented a much larger specific surface and, very likely, a different pore size distribution. In these conditions, a slightly different washing and elution protocols should be optimized. To address the non-selective adsorption of F observed in the case PD–C extracting serum, a more intense washing is probably needed in order to disrupt the non-selective adsorption of F. Additionally, increasing the acidic strength of the elution solvents might facilitate the release of phosphorylated analytes that exhibit the stronger interactions causing the incomplete elution of these analytes, as observed in the case of human plasma. This effect is likely due to the strong interaction between bis-imidazolium functional moieties resulted from monomer and the phosphorylated analytes within the microporous structure of PD–C. This effect is further magnified in the experiment with PD-E (Fig. 5e and f). In this case, the not augmented superficial area development on PD-E material resulted in an efficient washing of the non-phosphorylated F. On the other hand, the increased microporosity due to the etching step could be responsible for a stronger retention, thus resulting in an even more difficult desorption of the analytes. Given the importance of fractionation and the effective reduction of potentially interfering compounds in pharmacokinetic and biomarker analysis, PD–C offers promising prospects for achieving efficient separation and purification, thereby enabling enhanced sensitivity and accuracy in the analysis.

## Conclusions

In this study, three solid-phase extraction materials with different morphology, surface area, and pore size distribution were compared for their ability to enrich S1P and its active analogue FP from biological fluids. The adsorption isotherms demonstrated that Imid-co-DVB-based polymers significantly enhanced the total capacity compared to the previously reported polymers based on the more hydrophilic EGDMA cross-linker. This improvement was further confirmed through SPE experiments conducted on human sera and plasma samples, resulting in highly effective clean-up of the two phosphomonoesters. Further optimization of the extraction protocols is in progress, and we believe this will enhance the performance of the materials and will provide a valid tool to be adopted in bioanalysis for a fast and accurate quantification of the target S1P in clinical practice.

### Supplementary Information

Below is the link to the electronic supplementary material.

Supplementary file1 (DOCX 394 KB)
