# Supplementary material for: Enhanced selective capture of phosphomonoester lipids enabling highly sensitive detection of sphingosine 1-phosphate
Source: Anal Bioanal Chem. 2023 Sep 22;415(26):6573–82. doi: 10.1007/s00216-023-04937-8 (PMC10567913; doi:10.1007/s00216-023-04937-8)
Supplement: Supplementary file 1 — Supplementary file1 (DOCX 394 KB) [file 216_2023_4937_MOESM1_ESM.docx]

**Electronic Supplementary Material**

**Enhanced Selective Capture of Phosphomonoester Lipids Enabling Highly Sensitive Detection of Sphingosine 1-Phosphate**

*Giuliana Grasso,^1,2^ Eduardo M. Sommella,^1^ Fabrizio Merciai,^1^ Rahma Abouhany,^2^ Sudhirkumar A. Shinde,^2,3^ Pietro Campiglia,^1^ Börje Sellergren,^2^ and Carlo Crescenzi^1,*^*

^1^ Department of Pharmacy, University of Salerno, Via Giovanni Paolo II 132, 84084 Fisciano (SA), Italy.

^2^ Biofilm Research Center for Biointerfaces, Department of Biomedical Sciences, Faculty of Health and Society,

Malmö University, 23014 Malmö, Sweden.

^3^ School of Consciousness Dr. Vishwanath Karad, MIT World Peace University, 411038 Pune, India.

* Corresponding author: [carlo.crescenzi@unisa.it](mailto:carlo.crescenzi@unisa.it)

**Table S1:** List of compounds considered in competitive SPE experiments using 30 mg cartridges packed using PD-C. [*phospho-monoester lipids; **phospho-diester lipids; § without phosphorous.]

| **Lipids** | **class** | **Abbr.** | **M.W.**  **(g/mol)** |
| --- | --- | --- | --- |
| Sphingosine 1-Phosphate | * | S1P | 379.472 |
| Fingolimod Phosphate (FTY720-P) | * | FP | 387.457 |
| Fingolimod (FTY720) | § | F | 307.471 |
| 1,2-Dimyristoyl-sn-glycero-3-phosphorylglycerol (sodium salt) | ** | DMPG | 688.843 |
| 1,2-Dimyristoyl-sn-glycero-3-phosphocholine | ** | DMPC | 677.933 |
| 1,2-Dimyristoyl-sn-glycero-3-phosphoethanolamine | ** | DMPE | 635.853 |
| 1,2-Dimyristoyl-sn-glycero-3-phosphoserine (sodium salt) | ** | DMPS | 701.844 |
| 1,1’,2,2’-tetramyristoyl cardiolipin (sodium salt) | ** | CL | 1285.597 |

**
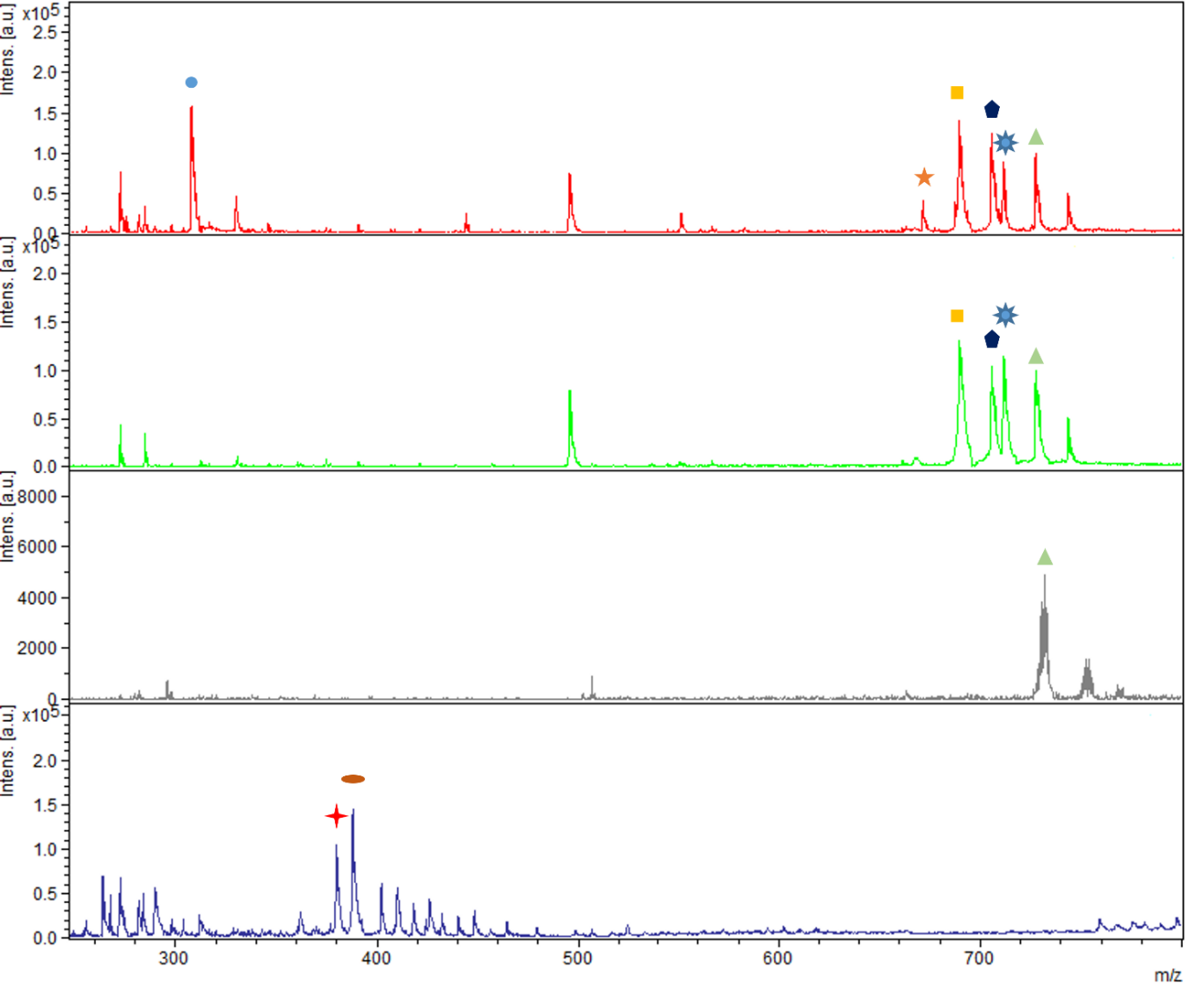
Figure S1:** MALDI MS recorded for phospholipids standards mixture of FT and W, and E SPE fractions after reconstitution in 100 µL of MeOH. Fingolimod (light blue circle), Sphingosine 1-Phosphate (red four-pointed star), Fingolimod Phosphate (brown oval), DMPE (orange star), CL (yellow square), DMPC (pentagon blue), DMPG (ten-pointed star), DMPS (green triangle).
